# A New Collaborative Knowledge-Based Approach for Wireless Sensor Networks

**DOI:** 10.3390/s100606044

**Published:** 2010-06-17

**Authors:** Joaquin Canada-Bago, Jose Angel Fernandez-Prieto, Manuel Angel Gadeo-Martos, Juan Ramón Velasco

**Affiliations:** 1 Telecommunication Department, University of Jaen, Alfonso X El Sabio 28, 23700 Linares, Jaen, Spain; E-Mails: jan@ujaen.es (J.A.F.-P.); gadeo@ujaen.es (M.A.G.-M.); 2 Department of Automatic, University of Alcala, Alcala de Henares, Madrid, Spain; E-Mail: juanramon.velasco@uah.es

**Keywords:** Wireless Sensor Networks, Fuzzy Rule-Based System, Cooperating Objects

## Abstract

This work presents a new approach for collaboration among sensors in Wireless Sensor Networks. These networks are composed of a large number of sensor nodes with constrained resources: limited computational capability, memory, power sources, *etc*. Nowadays, there is a growing interest in the integration of Soft Computing technologies into Wireless Sensor Networks. However, little attention has been paid to integrating Fuzzy Rule-Based Systems into collaborative Wireless Sensor Networks. The objective of this work is to design a collaborative knowledge-based network, in which each sensor executes an adapted Fuzzy Rule-Based System, which presents significant advantages such as: experts can define interpretable knowledge with uncertainty and imprecision, collaborative knowledge can be separated from control or modeling knowledge and the collaborative approach may support neighbor sensor failures and communication errors. As a real-world application of this approach, we demonstrate a collaborative modeling system for pests, in which an alarm about the development of olive tree fly is inferred. The results show that knowledge-based sensors are suitable for a wide range of applications and that the behavior of a knowledge-based sensor may be modified by inferences and knowledge of neighbor sensors in order to obtain a more accurate and reliable output.

## 1. Introduction

Over the past few years, Wireless Sensor Networks (WSNs) [1,2] have drawn the attention of multiple researchers. WSNs are defined [1] as networks composed of a large number of sensor nodes and can be conceived of as small computers with extremely basic interfaces and components. Each node consists of a processing unit with limited computational capability and memory, sensors, a communication device and a limited power source, usually in the form of a battery. Hence, WSNs have strong constraints on their energy resources and computational capacity. In addition, network protocols and algorithms must possess self-organizing capabilities that enable them to autonomously adapt to changes resulting from external interventions or requests submitted by an external entity.

As described in [1], sensor nodes can be used for continuous sensing, event detection, event identification, location sensing and local control of actuators. The range of applications of WSNs is very wide and includes intelligent agriculture, industrial control and monitoring, environmental monitoring systems, surveillance, health monitoring, traffic monitoring, *etc.*

Nowadays there is a persistent trend to include more and more functionalities into sensors in order to apply them in complex systems, to reduce the need for inter-sensor communication and to prolong battery lifetimes. In this sense, sensors with some intelligent capabilities have been proposed and several Soft Computing (SC) technologies have been applied to WSNs.

On the other hand, WSNs are perfect scenarios for sensor collaboration in order to obtain a global purpose. In this work, the term collaborative involves communication of individual measures among sensors to accomplish their tasks and to achieve a common goal.

Despite the wide interest in sensors and collaborative WSNs, little attention has been paid to collaborative Fuzzy Rule-Based Systems (FRBSs) for WSNs. FRBSs [3] are considered an extension of classical rule-based systems, because they deal with “IF-THEN” rules whose antecedents and consequents are composed of fuzzy logic statements (fuzzy rules) [4], instead of classical logic ones. One of the main characteristics of these systems is their capacity to incorporate human knowledge by accounting for its lack of accuracy and *uncertainty or imprecision*. FRBSs present some advantages over classical rule-based systems; for example: a) the key features of knowledge captured by fuzzy sets involve the handling of uncertainty and b) inference methods become more robust with the approximate reasoning methods employed within fuzzy logic.

This paper presents a new collaborative approach based on knowledge-based WSNs. The contributions of this work are as follows:

- We propose a knowledge-based sensor that executes a small FRBS that has been adapted to it and allows experts to incorporate uncertain and vague knowledge about the sensor data into the sensors;
- We define an application protocol that is adapted to sensor networks and enables the distribution of knowledge bases (KBs) and data communication between sensors;
- We examine the performance of the sensor and protocol, and observe from the results that the sensor reaction time and the network throughput and delay allow the system to be used in a wide set of applications;
- We propose a collaborative knowledge-based scheme that allows for collaboration among sensors in order to achieve a global network objective. The collaborative knowledge is defined by means of a FRBS, which is a very effective approach to support uncertainty and imprecision. As a real-world application, we present a knowledge-based WSN that concretely models a system of olive tree pests. The development of the olive tree fly is strongly related to the temperature and humidity conditions of the environment. The suitability of insecticide treatments applications should be evaluated if the risk of the plague appearance in an area surpasses a threshold level.

The remainder of the paper is organized as follows. The following section deals with related work and motivation. Section 3 shows the methodology that has been used: the structure of the collaborative knowledge-based WSN, the FRBS designed for the sensors, the main functions of the application protocol and the collaborative scheme. Section 4 shows the experimental results obtained related to the FRBS sensor and application protocol performance as well as a real-world application of collaborative FRBS sensors to modeling olive tree pests. Finally, conclusions are drawn in Section 5.

## 2. Related Work and Motivation

The introduction of some intelligent capabilities into sensor networks enables them to be applied to the control or modeling of complex systems. An intelligent sensor that modifies its internal behavior to optimize its ability to collect data from the physical world and to communicate it in a responsive manner to a host system was defined in [5]. Benoit *et al.* [6] presented a model of intelligent sensor systems and proposed three broad areas in which intelligence is applied to sensors: intelligence of perception, reasoning and social intelligence. Deckneuvel [7] reported an analysis of an intelligent sensor and proposed a language specifically developed for the design of such systems. As described in [8], a sensor network with intelligent behavior is a system that can adapt to the situation and present information that is relevant at the moment. Furthermore, Mekid [9] presented new structural concepts of intelligent sensors and networks with intelligent agents.

The past few years have witnessed a growing interest in intelligent sensors based on SC, such as in neural networks [10], fuzzy logic [11–15], evolutionary algorithms [16] and hybrid systems composed of fuzzy logic and neural networks [17]. Srinivasan *et al.* [18] presented a scheme for data-centric multipath routing in WSNs utilizing a fuzzy logic controller architecture at each node. The authors of [19] presented a distributed, general-purpose reasoning engine designed for WSNs. As described in [20], inference is one of the most common methods, techniques and algorithms applied in data fusion. Inference-based methods make decisions based on the system’s knowledge of the perceived situation. In [17], the term *“soft computing”* was introduced to the field of WSNs.

Recently more applications of SC in WSNs have been published: in [21] two intelligent localization schemes especially designed for WSN, in which SC technologies play a crucial role, are presented; in [22] a model to improve the distance estimation error between sensor is shown and in [23] the authors present a guideline on choosing the most optimal sensor combinations for accurate residential fire detection by mean of artificial intelligence techniques.

In [24] and [25] two real-world applications which use fuzzy inference systems in order to implement a collaborative approach have been described. These papers present two techniques based on fuzzy inference systems which are used to control the navigation of an autonomous mobile robot equipped with sensors.

Lately, several studies have been devoted to collaborative WSNs. Due to the characteristics of sensor networks, Gracanin *et al.* [26] argued that the nodes used in their work have to collaborate in order to accomplish their tasks: sensing, signal processing, computing, routing, localization, security, *etc*. Moreover, in [27], P. J. Marrón claimed that “... recently the idea of WSNs has started to appear, where entities that sense their environment not only operate individually, but collaborate together using *ad hoc* network technologies to achieve a well-defined purpose of supervision of some area, some particular process, *etc.*” In both [27] and [28], a cooperating object (CO) was defined as a single entity or a collection of entities consisting of *sensors*, *controllers (information processors)*, *actuators* or *cooperating objects* that communicate with each other and are able to achieve, more or less autonomously, a common goal. As described in [28], wireless sensors and actuator networks are typical examples of such cooperating objects. These networks consist of objects that are individually capable of simple sensing, actuation, communication and computation, but the full capabilities of such networks are reached only by the cooperation of all these objects.

All the above works are related and relevant to: sensors which incorporate intelligent capabilities, sensors based on SC technologies, collaborative sensors and sensors as cooperating objects. As indicated in these works, an improvement in the reliability, responsiveness and accuracy of the sensors behavior can be achieved in WSNs if they incorporate collaborative algorithms. Nevertheless, they require a significant amount of resources in terms of CPU performance, memory, wireless communication bandwidth and battery consumption.

In order to solve this problem, a fuzzy rule-based collaborative approach (D-FLER) has been presented in [19]. This system incorporates distributed and embedded collaborative mechanisms of reasoning on the observed data and taking decisions or actions in a coordinated manner. Following this idea, D-FLER uses two types of inputs: individual observations (sensor readings of the current node) and neighborhood observations (fuzzified sensor data from the neighboring nodes). However, D-FLER presents some limitations, derived from the need to:

- Transmit a significant amount of data to neighbor nodes.
- Process a significant amount of data.

These features cause a decrease in the lifetime of the batteries.

To improve the above-mentioned problem, in this work we propose a new scheme that fuse:

- Individual observations.
- The knowledge that was obtained from their neighbor nodes.

To the best of our knowledge, the collaborative approach for WSNs presented in this paper is the first one that proposes the use of a FRBS to define the collaborative knowledge. This approach allows the users to define the collaboration among sensors by means of a specific KB (variables, fuzzy sets and rules), which presents significant advantages:

- The collaborative scheme may deal with uncertainty and imprecision.
- It is possible to separate control or modeling knowledge from collaborative knowledge, using interpretable rules in both cases.
- The collaborative approach may support sensor failures and communication errors because, in this case, the collaborative sensor would infer a proportional value to the number of failures between control or modeling knowledge (local knowledge without collaborative scheme) and the collaborative scheme.

Some scenarios that could benefit from the proposed approach are real-world applications with complex control or modeling in which sensor inferences may be affected by measures or inferences of neighboring sensors, e.g., event detections (fire detection, alarms), environmental monitoring (water quality, pest detection), industrial process control, robotics, etc.

## 3. Collaborative Knowledge-Based WSN Proposal

Despite the propensity for highly constrained resources (microcontroller, memory, battery, communications, *etc*.) in sensors and sensor networks, there is a persistent trend to include more and more functionalities into sensors both to reduce the need for inter-sensor communication and to prolong battery lifetimes. Resource constraints make it impossible to directly utilize the traditional schemes or approaches of FRBSs and Evolutionary Algorithms (EAs) in the case of sensor systems. Instead, it is necessary to develop a new scheme by which sensors can execute a small knowledge-based system of functions, leaving other functions to be executed in a system with greater information processing capacity. The current information processing capacity of sensors is low due to the microcontroller and the small amount of memory that such devices usually contain.

Sensors do not have man-machine interfaces to enable users to update sensor data or to view the evolution of a particular variable. However, sensor networks do permit sensors to share data and knowledge; knowledge updates and sensor-to-sensor collaboration can support the attainment of global objectives. An objective of this work is to design a FRBS that can be executed in a sensor and that includes a collaborative scheme for sharing variables, data and knowledge. What follows is a discussion on the structure of the proposed system, the design of the FRBS, the main features of the application protocol and the collaborative scheme by which information is shared by sensors as well as proposed methods to share local and remote information.

### 3.1. Structure of the System

The proposed system was designed to separate some functions that are traditionally integrated into the inference engine of a FRBS, so as to allow sensors to execute a knowledge-based system.

The system is composed of a computer, an access point, a sensor network, a FRBS adapted for execution in the sensors, a communication protocol and a collaborative scheme. The main functions of the components are as follows:

- Computer: edit KBs using linguistic labels (variables, fuzzy sets and rules), access the sensor network, communicate with sensors, monitor sensor state, *etc.*
- Sensor network: allow sensors and computer to communicate. The network consists of an access point and a set of sensor nodes with sensing capability, data processing and communicating capabilities.
- FRBS adapted to the sensors: infer the output by means of an inference engine and knowledge about the system.
- Application protocol: allow the elements of the system to communicate data and knowledge.
- Collaborative scheme: share and fuse data and knowledge between sensors and computer.

### 3.2. Fuzzy Rule-Based System Adapted to a Sensor Node

The knowledge-based sensor is based on the structure of the Mandani FRBS, which consists of the following components [3]:

- A KB, which stores knowledge about the problem. The KB is composed of a Data Base (DB), which contains the linguistic term sets considered in the linguistic rules, and a fuzzy control Rule Base (RB), which comprises the collection of linguistic rules representing expert knowledge in the following form: $\begin{array}{ccc} \text{IF}\;\;{\text{X}}_{1}\;\;{\text{is A}}_{1}\;\;\text{and}\ldots \text{and}\;{\text{X}}_{\text{n}}\;\text{is}\;{\text{A}}_{\text{n}} & \text{THEN} & \text{Y is B} \end{array},$where X_i_ are input variables, A_i_ are fuzzy sets related to the input variables, Y is the output variable and B is a fuzzy set related to the output variable.
- A fuzzy inference engine is composed of the following:
  - A fuzzification interface, which transfers the values of the input variables into fuzzy information, assigning grades of membership to each fuzzy set defined for that variable;
  - An inference system, which infers fuzzy actions by means of fuzzy implications and the rules of inference of fuzzy logic;
  - A defuzzification interface, which yields a non-fuzzy control action from an inferred fuzzy control action. This interface aggregates the information provided by the output fuzzy sets to obtain an output value from them.

Due to sensor constrains, we introduced several modifications to the structure of Mandani FRBS, and utilized technologies requiring the least computational burden in order to minimize computational cost and battery consumption.

To reduce the computational burden, we propose the following:

- An approach in which sensor nodes execute a small, but complete, FRBS;
- Only triangular or trapezoidal fuzzy sets be made available, which decreases the number of operations executed in the inference process;
- The input and output interfaces only admit linear conversions;
- A First Infer Then Aggregate (FITA) inference approach be used;
- Experts define the knowledge by means of linguistic labels, variables and rules, editing a KB in the computer. The KB is then translated into appropriate numerical values that are transmitted to the sensors.
- The inference engine work with numerical values of variables, fuzzy sets and rules instead of linguistic labels. The translation of linguistic labels is executed in the computer, so the sensor nodes do not have to do this task;
- The number of fuzzy sets defined in each variable be small. Although there is not a direct relationship between the number of fuzzy sets defined in each variable and the inference time, an excessive number of fuzzy sets would involve a large number of rules, and as a consequence, the inference time would increase.

To reduce the battery consumption and prolong the battery lifetime, we propose that:

- Sensors operate in a work cycle in which they first infer the output, and then are configured in a sleep mode;
- Sensors only transmit remarkable outputs to their neighbors (e.g., when an inferred alert surpasses a threshold).

**Figure 1.**
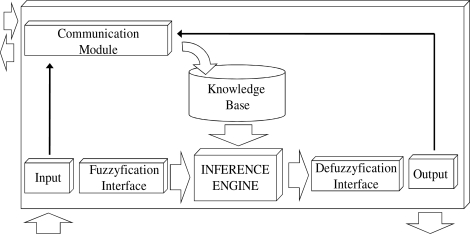
Structure of the knowledge-based sensor.

Figure 1 shows the structure of a FRBS sensor that consists of a communication module, input and output interfaces with scaling functions, fuzzification and defuzzification interfaces, a KB and an adapted inference engine.

The communication module mainly allows the sensor to update its KB and communicate the results of its inferences to other sensors. Other functions are described in Section 3.3.

The input and output interfaces obtain and translate crisp values into the interval [0,1] using a linear conversion, and the fuzzification and defuzzification interfaces establish the correspondence between the normalized values of the variables and fuzzy sets defined on the universe of discourse.

The KB, composed of data and rule bases, includes variable definitions, fuzzy sets defined for each variable and rules. After the variables are defined, fuzzy sets are associated to each variable. Each fuzzy set is defined by means of only four real numbers, enabling the system to utilize triangular or trapezoidal shapes. The KB includes a set of IF- THEN rules in which every rule has an antecedent, composed of several propositions, and one consequent. Every antecedent preposition and the consequent relate a variable and a fuzzy set defined in the variable. The adapted inference engine infers the system output by means of the inputs and the KB, taking into account those modifications that have been previously proposed.

### 3.3. Application Protocol

The application protocol is designed to transfer data and KBs between the computer and sensors and among sensors using transport layer services. Although KBs are usually edited in the computer, transmitted to sensors and executed in sensors, it is also possible for information generated in sensors (e.g., values of inferred output) to be sent to other sensors or the computer.

In order to reduce battery consumption, we propose that the application protocol work incrementally (*i.e.*, the complete KB is first sent from the computer to the sensors, only after which can a part of the KB (variables, fuzzy sets, *etc.*) be updated) and that the protocol allow the sensors to operate in a sleep mode.

The main functions of the communication protocol are the following:

- Transmit elements of a DB to sensors, including the number of variables in the KB, number of fuzzy sets in each variable, the fuzzy set associated with each variable, the type of each variable (local or remote) and the data range of each crisp value;
- Transmit elements of a RB to sensors, including the number of rules, number of propositions in the antecedent as well as the antecedent and consequent themselves;
- Transmit values of input and output variables among sensors or between a sensor and the computer.
- Perform operations such as add, update, delete or activate a KB; define the interval between inferences and remain in sleep mode or in active mode between inferences.
- Register the state of system variables, so as to register a variable and obtain the value of variables in order to represent them in the computer, *etc*.

The protocol allows the user to change the knowledge of the system in real time, to incorporate different knowledge in different sensors and to verify the evolution of some variables in the system.

The main aspects of the implementation of the application protocol are the following:

- The computer and every sensor have a communication agent, which has been developed in Java;
- The application protocol utilizes transport layer services which are available in WSNs (connection-oriented, connectionless and broadcast);
- An Application Protocol Data Unit (APDU) has been defined to support the protocol commands and responses between nodes (computer and sensors). In this work, the following commands have been utilized:
  - KBs transmission. Based on a connection-oriented service, this command allows the computer to send a complete KB to the sensors.
  - Variable values transmission. This command allows the sensor to send the value of an input variable or the value of an inference.
  - Work cycle. The sleep mode interval between inferences is sent from the computer to sensors using a connection-oriented transport service.
  - Alarm notification. Based on a connectionless broadcast service with only one hop, each sensor can notify remarkable variable values to neighbor sensors (e.g., when a variable surpasses a threshold).

### 3.4. Collaborative Scheme

In Section 3.2, we described a system in which every sensor has an FRBS including a KB. This knowledge, which may differ for each sensor, allows every sensor to infer its local outputs; it may thus be considered local knowledge. On the other hand, sensor networks and knowledge-based systems are well poised to take advantage of collaboration and data and knowledge sharing between elements of the systems (sensors and computer) to support global objectives.

Each sensor is able to share its data (e.g., the value of a local variable) and/or knowledge (e.g., a fuzzy set) with another sensor, with a group of sensors or with all the elements of the system. Therefore, it is conceivable that a sensor may have local and remote data: local data being that obtained directly by the sensor by means of its own resources (e.g., measurements of temperature) and remote data being that obtained by others sensors and subsequently transmitted to the given sensor. The same occurs sharing scenario with knowledge; that is, sensor may have local and remote knowledge. Local knowledge allows the sensor to infer its local decision, while remote knowledge complements the local knowledge to promote a global objective.

This section presents a scheme for collaboration among knowledge-based sensors to obtain a shared objective. The subsections that follow reveal what information may be shared and discuss different schemes to incorporate remote data and knowledge.

#### 3.4.1. Information to be Shared by Sensors

Every knowledge-based sensor has a set of input variables, a KB and an output variable whose value is obtained by its inference engine. Due to the structure of the network and the application protocol, it is possible for sensors to share the following information with the rest of the system in order to obtain a global objective: shared data may include the values of input variables, the value of the output variable, fuzzy sets, variable definitions and rules.

Sensors may share the value of variables, which are locally obtained, with other sensors by sending it through the network. Therefore, the values of the input variables for a sensor node may be locally or remotely obtained and represent the values of the antecedent variables of its knowledge.

**Figure 2.**
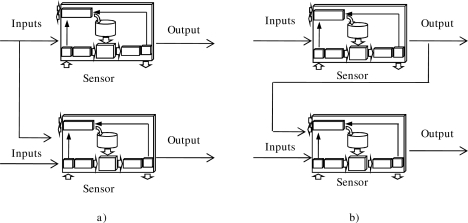
Sharing input and output variables between two sensors.

Figure 2 a shows a sensor sharing an input variable. In this scenario a sensor obtains the value of a variable (e.g., the sensor measures the temperature) and transmits this value to other sensors, which can utilize it to infer their output.

Another possibility is to share the value of an output variable of a sensor (Figure 2b). This value is determined by the local knowledge of the sensor and that information inferred by the sensor. In this case, the shared value takes into account the transmitting sensor’s knowledge; thus, it is a way to share knowledge.

EAs could be applied to sensors’ local knowledge. The constrained resources of the sensors restrict application of the EA to tuning the points of fuzzy sets or scale functions in order to improve their behavior. Therefore, elements that undergo tuning, such as fuzzy sets and scaling functions, could be shared with the rest of the sensors.

#### 3.4.2. Integration of Data and Knowledge

In the proposed collaborative knowledge-based WSN, each sensor can base its inferences on local or remote data and knowledge. This section proposes different ways to integrate local and remote information.

Local data are the values of variables that are locally obtained by the sensor, whereas remote data are the values of variables that are obtained or inferred by another sensor. Similarly, local knowledge refers to the KB (variables, fuzzy sets and rules) that is locally utilized by a sensor in order to infer the value of an output variable, while remote knowledge refers to knowledge that may utilize local and remote variables in order to promote a global objective.

The sensor network and the application protocol allow the sharing of data and knowledge, so, if remote information is available, each sensor can base its inferences on local information (data and knowledge) and remote information (data and knowledge). However, if remote information is not available, each sensor can only base its inferences on its local information.

**Figure 3.**
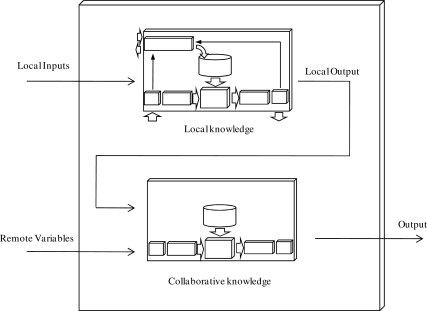
A sensor with local and collaborative knowledge.

The following methods are suggested to fuse local and remote information (data and knowledge):

- Utilize only a KB, which incorporates local and remote information. Remote variables and rules are added to the KB so that the rules are able to use external data in their inferences. The inference engine will then take into account those remote rules if remote information is available.
- Incorporate a complete collaborative KB (Figure 3). In this method the sensor utilizes a KB with local information (data and knowledge) and another complete KB with collaborative information (remote data and knowledge). In this case, the local output and remote variables represent inputs to a second FRBS. After inferring the output based on the local knowledge base, the inference engine will take into account the collaborative KB, which makes available all the external information. This method presents additional advantages: the number of rules is smaller and the rules are more interpretable because of the two KBs.
- Finally, the third option is to design a hierarchical system (using several KBs) in which local and remote data and KBs can be mixed.

A common feature of all three methods is the use of a FRBS to fuse local and remote information; this feature is very effective in an environment with uncertainty and imprecision.

## 4. Experimental Results

This section presents the results of several experiments carried out to measure the performance of the knowledge-based sensor and the application protocol. Subsequently, an evaluation of the collaborative scheme is presented in order to show the effects of collaborative FRBS sensors on a modeling system for outbreaks of olive tree pests. The collaborative knowledge-based approach proposed here follows from our previous works [29,30], in which we presented the performance of a preliminary FRBS sensor node without the collaborative scheme.

### 4.1. Knowledge-Based Sensor Performance

The knowledge-based sensor has been designed and developed for Sun SPOTs [31] sensors, which have the following main characteristics: they can execute Java programs (J2ME), 180 MHz, 32-bit ARM920T CPU, 512 K RAM, 4M Flash, 3.7 V 720 mAh battery, three modes (awake: draw current 86 mA; shallow sleep: 24 mA; deep sleep: 32 μA), integrated sensors (3-axis accelerometer, temperature, light), several inputs and outputs (analog and digital), and a IEEE 802.15.4 compliant radio to perform wireless communication. Routing, meshing and fragmentation with the Sun SPOT stack are accomplished through the LowPAN protocol. Multi-hop connectivity is accomplished by AODV, a sophisticated routing protocol for *ad hoc* networks. In the transport layer, the protocols that allow Sun SPOT applications to access the network are the RadioGram protocol and the RadioStream protocol.

In order to evaluate the performance of the knowledge-based sensor, we utilized a testbed comprising a WSN with 10 Sun SPOTs, a base station (access point) and a personal computer connected to the Internet. Several experiments were conducted, including the use of a preliminary FRBS adapted to the Sun SPOT sensor [30] to study the relationship between the number of inferences per second and the number of rules in the KB, as well as the relationship between the number of inferences per second and the number of antecedent propositions per rule.

In this work, the reaction time of the knowledge-based sensors was measured, taking into account that the KB we used has 25 rules, with two propositions in the antecedent per rule (Table 1); it is possible to reduce the KB to 14 rules because of redundant knowledge; the collaborative KB has 9 rules with two propositions in the antecedent per rule (Table 2). The reaction time of the sensor (*i.e.*, the time to execute both KBs) was 5.7 ms, which agrees with previous work and is sufficient for the real-world application presented in this work.

Taking into account the time to execute both KBs (5.7 ms in awake mode, with a current draw of 86 mA), the interval between inferences (30 min in deep sleep mode, drawn current = 32 μA) and the battery capacity (720 mAh), we estimate that the lifetime of the batteries in this system to be around 608 days.

In addition, we carried out a battery consumption test on the Sun SPOT sensors of the testbed. First, the battery was completely charged. Next, the sensor followed a cycle composed of an inference of both KBs and an interval of 30 s between inferences in deep sleep mode. The capacity of the sensor battery was transmitted to the computer every 30 min for post analysis. The test ended when the battery was completely discharged. In these conditions, the average power consumption was 0.0218 mAh/cycle; the estimated lifetime of the battery is around 688 days, taking into account a cycle every 30 min.

### 4.2. Application Protocol Performance

In the previous section, we showed that the sensors are able to execute the FRBSs that have been adapted to them. In this section, the aim is to measure the main characteristics of the distribution of the KB among the sensors of a WSN by means of the application protocol described in Section 3.3.

This application protocol has been implemented in the 2.33 release of the network simulator ns2 [32] and configures the network and nodes with the same characteristics as the Sun SPOT network and nodes to measure the delay and throughput of the KB distribution.

The WSN was configured with 25 sensors uniformly scattered in a 100 m by 100 m area, with the PAN coordinator located in the center. The size of the KB was 340 Bytes. The nodes were configured using the transport protocol RadioStream (simulated using TCP), the routing protocol for the *ad hoc* network AODV, a link and MAC layer IEEE 802.15.4 (2.4 GHz), a transmit power of 0 dBm, a receiver threshold of −77 dBm, and a receiver sensitivity −90 dBm. During the simulation, the PAN coordinator sent the KB to the sensors sequentially.

In these conditions, the time to distribute the KB to all sensors was 33 s, and the throughput remained around 15 KB/s (120 Kbps) during the simulation. This distribution time is sufficient for the real-world application presented in this work; sensors can only transmit the results of their own inferences to neighboring sensors after the initial distribution.

### 4.3. Evaluation of the Collaborative Scheme

In order to show the effects of the collaborative scheme on each sensor, a model system for olive tree pests is given as a real-world application of the collaborative knowledge-based WSN. We consider this application as a case of FRBS modeling instead of FRBS control since there is no real time action that affects the input variables. However, it would be possible to evaluate the suitability of insecticide treatments on the trees.

The development of the olive tree fly is strongly related to the temperature and humidity conditions of the environment. In real world, it is possible to observe isolated or grouped areas with high concentrations of flies; experimental observations have shown that the concentration of these areas increases the risk of degradation of olive trees. For this reason, the risk of the appearance of a plague in an area will increase if the risk of plague also increases in a proximal area.

In the proposed system, each sensor collaborates with neighboring nodes to produce a more accurate and reliable alert status. Each node has an embedded cooperative algorithm (a collaborative FRBS) that uses local observations to infer its local output and the perceptions from its neighbor’s alert to infer the alert status of its area.

**Figure 4.**
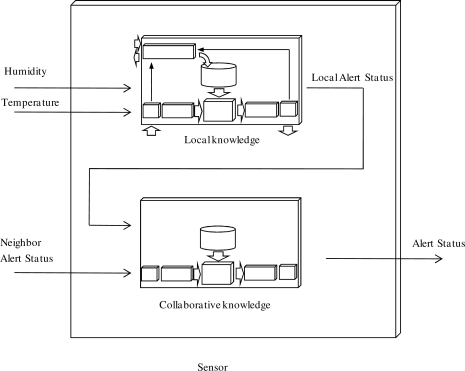
Inner structure of collaborative knowledge-based system.

To facilitate the model, a collaborative system composed of two FRBSs was embedded in each sensor (Figure 4). The first FRBS has two inputs: the local humidity and temperature; and one output: the local alert status. The second takes this local alert status in addition to the neighbors’ alert statuses as inputs, and produces the alert status as output. If the alert status produced surpasses a certain threshold (e.g., 0.75), indicating a risk of pest appearance, the suitability of insecticide treatments applications should be evaluated.

**Figure 5.**
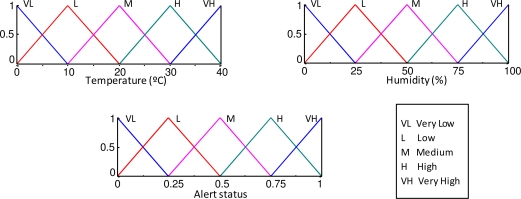
Membership functions of the inputs and output variable fuzzy sets (local knowledge).

The KB that generates the local alert status (local knowledge) is composed of two input variables (temperature and humidity), one output fuzzy variable (local alert status), membership functions (Figure 5) and the RB (Table 1).

**Figure 6.**
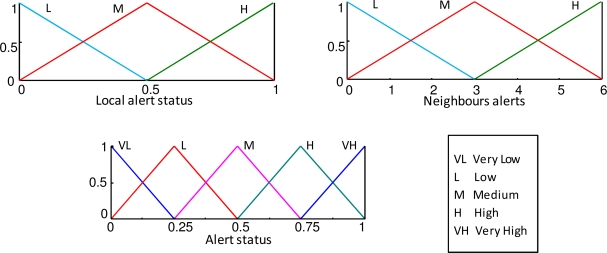
Membership functions of the inputs and output variables fuzzy sets (collaborative knowledge).

The KB that generates the alert status (collaborative knowledge) is composed of two input variables (the local and neighbor’s alert status), one output fuzzy variable (alert status), membership functions (Figure 6) and the rule base (Table 2).

The collaborative scheme was developed in Java (J2ME) for embedding in a Sun SPOT sensor. However, since the objective of this experiment is to evaluate the effects of the collaborative scheme on the sensor model surface, a wide set of system states, defined by the values of the temperature, humidity and neighbor alert status, is needed to generate the surfaces. Therefore, this experiment was carried out on a computer (with the same collaborative scheme designed for the sensors) in order to facilitate manipulation of the values of the variables.

**Figure 7.**
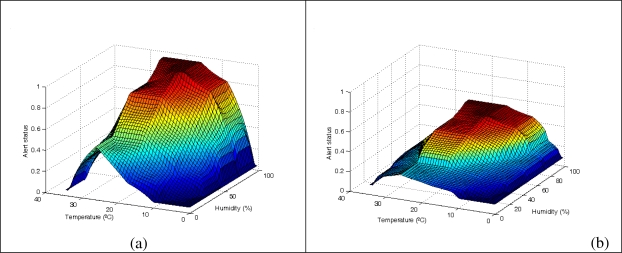

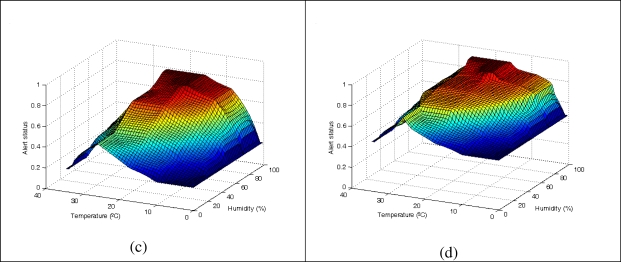
Input-output model surface for a single knowledge-based (a) and collaborative knowledge-based system, with a low (b), medium (c) and high (d) value of the number of neighbor sensors with an alert status above 0.75.

In order to test the viability of the proposed approach, a comparative analysis of the sensor behavior is shown for four different situations: sensor nodes with a local knowledge only-based system (Figure 7a), sensor nodes with a collaborative knowledge-based system with low (Figure 7b), medium (Figure 7c) and high (Figure 7d) values of the number of neighbor sensors with an alert status above 0.75.

Note the following observations about the control surfaces:

- Figure 7a presents a high risk/peak value and decreases quickly, so it surpasses the threshold level only in areas of low humidity and temperature.
- Figure 7b presents a low risk/peak value, and does not surpass the threshold level.
- Figure 7c presents a medium risk/peak value and decreases quickly, so it surpasses the threshold level only in an area of very low humidity and temperature.
- Figure 7d presents a high risk/peak value and decreases slowly, so it surpasses the threshold level in a large area of humidity and temperature.

As can be observed in Figures 7 b–d, the collaborative FRBS sensor adapts its behavior (*i.e*., its model surface) depending on the number of neighbors that have detected the possibility of pest appearance, increasing or decreasing its own alert status depending on neighbor’s alert status.

## 5. Conclusions

This work has presented an effective approach to collaboration in knowledge-based WSNs in which sensors can execute a small FRBS, share variables, data and knowledge, and collaborate in order to achieve a global objective by fusing local and remote information. In addition, a real-world application of the collaborative FRBS scheme has been shown in order to model outbreaks of pests among olive trees.

The results show that the behavior of a sensor may be modified by the knowledge of its neighbor sensors. Such sensors take into account not only their own knowledge but also the inferences and knowledge of neighbor sensors in order to obtain an output that is based on a wide set of measures. In this real-world application, the alarm status of individual sensors is modified by a knowledge-based system that incorporates the local alarm status and the alarm status of neighbor sensors.

Moreover, the experiments have shown that the reaction time of the knowledge-based sensor is more than sufficient for this real-world application. Additionally, the throughput and delay obtained in the network indicate that it is possible to transmit KBs (from the computer to each sensor) in a short time, taking into account that the complete KB is transmitted once before sensors operate. Afterwards, only the values of significant variables are transmitted.

These results suggest that the collaborative scheme for FRBS-embedded sensors produces a more accurate and reliable alert status than a single knowledge-based system; moreover, this scheme can be utilized in a wide range of applications in which there is expert knowledge coupled with uncertainty and imprecision.

## Figures and Tables

**Table 1.** Base of rules used in the local FRBS.

*Alert*	***Temperature***					
***Humidity***		VL	L	M	H	VH
	VL	VL	VL	L	M	VL
	L	VL	L	M	M	VL
	M	VL	M	H	H	L
	H	VL	H	VH	H	L
	VH	VL	H	VH	VH	L

VL: Very Low; L: Low; M: Medium; H: High; VH: Very High

**Table 2.** Base of rules used in collaborative knowledge.

*Global Alert*	***Neighbor Alerts***			
***Local Alert***		L	M	H
	L	VL	L	M
	M	L	M	H
	H	M	H	VH

VL: Very Low; L: Low; M: Medium; H: High; VH: Very High
